# Divalent cations in human liver pyruvate kinase exemplify the combined effects of complex-equilibrium and allosteric regulation

**DOI:** 10.1038/s41598-023-36943-2

**Published:** 2023-06-29

**Authors:** Tyler A. Martin, Aron W. Fenton

**Affiliations:** Department of Biochemistry and Molecular Biology, The University of Kansas Medical Center, MS 3030, 3901 Rainbow Boulevard, Kansas City, KS 66160 USA

**Keywords:** Biochemistry, Biophysics

## Abstract

There is growing recognition that the functional outcome of binding of an allosteric regulator to a protein/enzyme is influenced by the presence of other ligands. Here, this complexity is exemplified in the allosteric regulation of human liver pyruvate kinase (hLPYK) that is influenced by the presence of a range of divalent cation types and concentrations. For this system, fructose-1,6-bisphosphate (activator) and alanine (inhibitor) both influence the protein’s affinity for the substrate, phosphoenolpyruvate (PEP). Mg^2+^, Mn^2+^, Ni^2+^, and Co^2+^ were the primary divalent cations evaluated, although Zn^2+^, Cd^2+^, V^2+^, Pb^2+^, Fe^2+^, and Cu^2+^also supported activity. Allosteric coupling between Fru-1,6-BP and PEP and between Ala and PEP varied depending on divalent cation type and concentration. Due to complicating interactions among small molecules, we did not attempt the fitting of response trends and instead we discuss a range of potential mechanisms that may explain those observed trends. Specifically, observed “substrate inhibition” may result from substrate A in one active site acting as an allosteric regulator for the affinity for substrate B in a second active site of a multimer. We also discuss apparent changes in allosteric coupling that can result from a sub-saturating concentration of a third allosteric ligand.

## Introduction

In the study of allosteric systems, it is becoming increasingly recognized that multiple ligands can simultaneously interact with a protein to collectively result in different regulatory outcomes compared to the outcome associated with a single effector binding. For example, the maximum extent of change in substrate affinity elicited by one allosteric effector can be modified by the presence of a second effector. We can also envision an example in which an allosteric activator of substrate affinity can act as an allosteric inhibitor when a second effector is present. Characterizing proteins that bind multiple ligands can be further challenged when the small molecules interact with each other in a complex equilibrium system. In this study, human liver pyruvate kinase (hLPYK) was used to demonstrate enzymatic responses possible in a system that combines allosteric regulation and complexed equilibrium.

Pyruvate kinase (PYK) has long been studied as a model of allosteric regulation^1–5^. This homotetrameric enzyme catalyzes the transfer of a phosphate moiety from the substrate, phosphoenolpyruvate (PEP) to ADP in the final reaction in the glycolytic pathway^6^. The resulting products are pyruvate and ATP. Among the four mammalian PYK isozymes, hLPYK and the isozyme expressed in mature red blood cells (RPYK) are two products from the same gene^7,8^. hLPYK is allosterically activated by fructose-1,6-bisphosphate (Fru-1,6-BP), which alters the affinity for the PEP substrate^9–11^. While alanine is commonly studied as an allosteric inhibitor of hLPYK^11,12^, other small amino acids also regulate the enzyme^13^. It is conceivable that cellular regulation of PYK is the result of the combined concentration of all amino acids with the final activity being determined by the competitive binding of many amino acid types and the amino-acid-specific quantitative influences on PEP substrate affinity.

**Figure 1:**
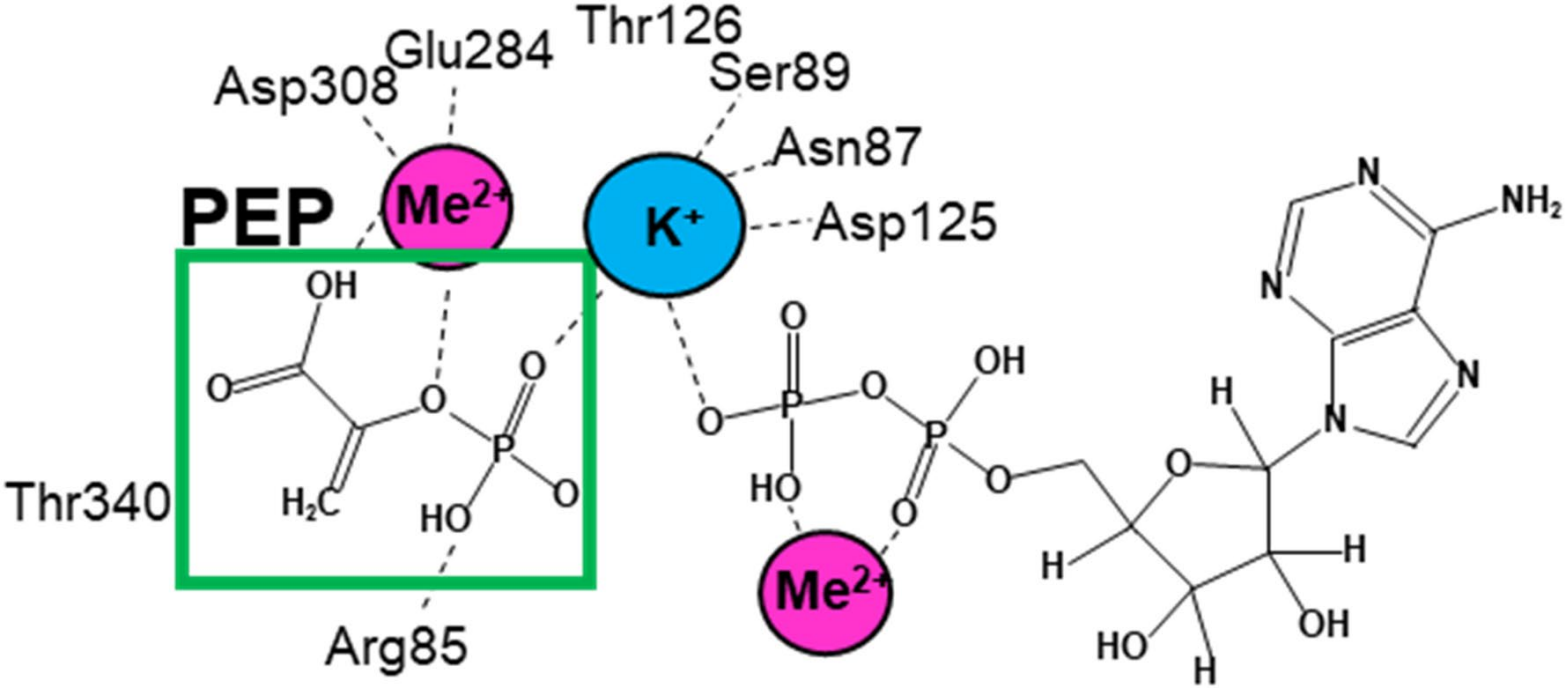
A cartoon depiction of possible coordination of substrates in the active site of PYK. These possible interactions are drawn from structural data of co-crystalized PYK proteins with various substrate analogs present in the active site^18–20,22,23^. The focus here is on PEP, the substrate that experiences modified substrate affinity in response to allosteric effectors. Across the available structures, Thr340 hovers just beyond a distance that is considered to make a direct bond with the bound PEP. K^+^ may serve to interact with one or both substrates at various steps in the catalytic cycle. There are surprisingly few direct interactions between the allosteric responding PEP substrate and amino acids from the protein. Instead, PEP primarily binds via bonding to the protein-bound divalent cation (the “protein-bound divalent cation”) and the monovalent cation.

PYK isozymes were the first enzyme to be identified as requiring both a monovalent and a divalent cation for activity (Fig. 1)^6,14–16^. Further research has revealed that PYK activity requires divalent cations at two different binding sites^16–20^. One divalent cation forms a divalent-ADP complex that serves as a substrate in the active site. The second is a protein-bound divalent cation that interacts directly with the PEP substrate. In addition, many isozymes (including hLPYK) require a monovalent cation for activity. However, some non-mammalian PYK isozymes position the side-chain charge of a lysine residue to function in place of the monovalent, making those isozymes monovalent-independent^14,21^. The monovalent cation (or lysine functional group) is located at the interface of the two substrates in the active site, such that it forms bonds to the transferred phosphate.

A curious feature of the PYK active site is its limited direct contact with the allosteric responding substrate, PEP (Fig. 1). Most available structures indicate only one side-chain interaction and that interaction is with the oxygen that bridges the carbon backbone of PEP and the leaving phosphate group. Other interactions with PEP occur indirectly via the protein-bound divalent cation and the monovalent cation. Therefore, it is relevant to ask whether allosteric activation of hLPYK by Fru-1,6-BP and allosteric inhibition by amino acids affect PEP affinity by changing bonding interactions with the monovalent and divalent cations. An alternative option, PEP affinity can conceivably be modified by adding or removing interactions that contribute negatively to binding energies, such as modulating a charge repulsion that does not directly interact with the bound substrate.

A previous study explored how a range of monovalent cation types and concentrations modulate allosteric coupling between PEP and Fru-1,6-BP and between PEP and alanine^14^. That previous study concluded that the active site monovalent cation does not likely contribute to the mechanism of allosteric regulation by either Fru-1,6-BP or by alanine. A previous study also reported the quantitative differences in the Fru-1,6-BP and alanine allosteric outcomes for hLPYK when Mg^2+^ vs. Mn^2+^ was added as the divalent cation^14^. The influence of these two divalent cations on allosteric regulation in yeast PYK has also been extensively studied^24–27^.

In this work, we extend the characterization of the influence of the divalent cation type and concentration on the allosteric coupling in hLPYK. Divalent cations are known to interact directly with PEP, ADP, Fru-1,6-BP and Ala in solution. Therefore, observed data trends may result from allosteric regulation, complexes between small molecules, and or both. We extensively discuss a range of mechanisms that can result in data trends exemplified by the data generated for this hLPYK system.

## Results and discussion

### An empirical evaluation of response features

At the onset of this study, we recognized that the system is inherently a multiple-equilibrium system. It has been well established that ADP, PEP, Fru-1,6-BP, and alanine interact directly with divalent cations in solution, irrespective of their interactions with the hLPYK protein^28–34^. We do not have complete knowledge of all possible binding interactions among the small molecules used in this study, particularly those involving higher-order interactions between different types of complexes. Additionally, buffers are known to have varying affinities for divalent cations^35,36^, and contaminating phosphate that is, present in several of the added small molecules can serve as a V-type activator of hLPYK^37^ and a binder of divalent cations. This makes determining the concentrations of free ligands a challenging task. As a result, our study was designed to generate data trends and use those trends to discuss potential explanations for each response feature, rather than obtaining binding values from the generated data. It is important to note that multiple mechanisms may contribute simultaneously to observed data trends. For instance, the formation of small molecule complexes may sequester small molecules, preventing them from interacting with the protein or binding. Those same small molecule complexes could bind to the protein competitively with a non-complexed small molecule or binding at a unique site on the protein to elicit a new response. Therefore, even before considering other (e.g. allosteric) potential mechanisms, the complex equilibrium associated with this system offers several potential explanations for data trends. (For a list of potential binding reactions, see the supplemental Table 1).

### Activity response to increasing divalent cation concentration

**Figure 2:**
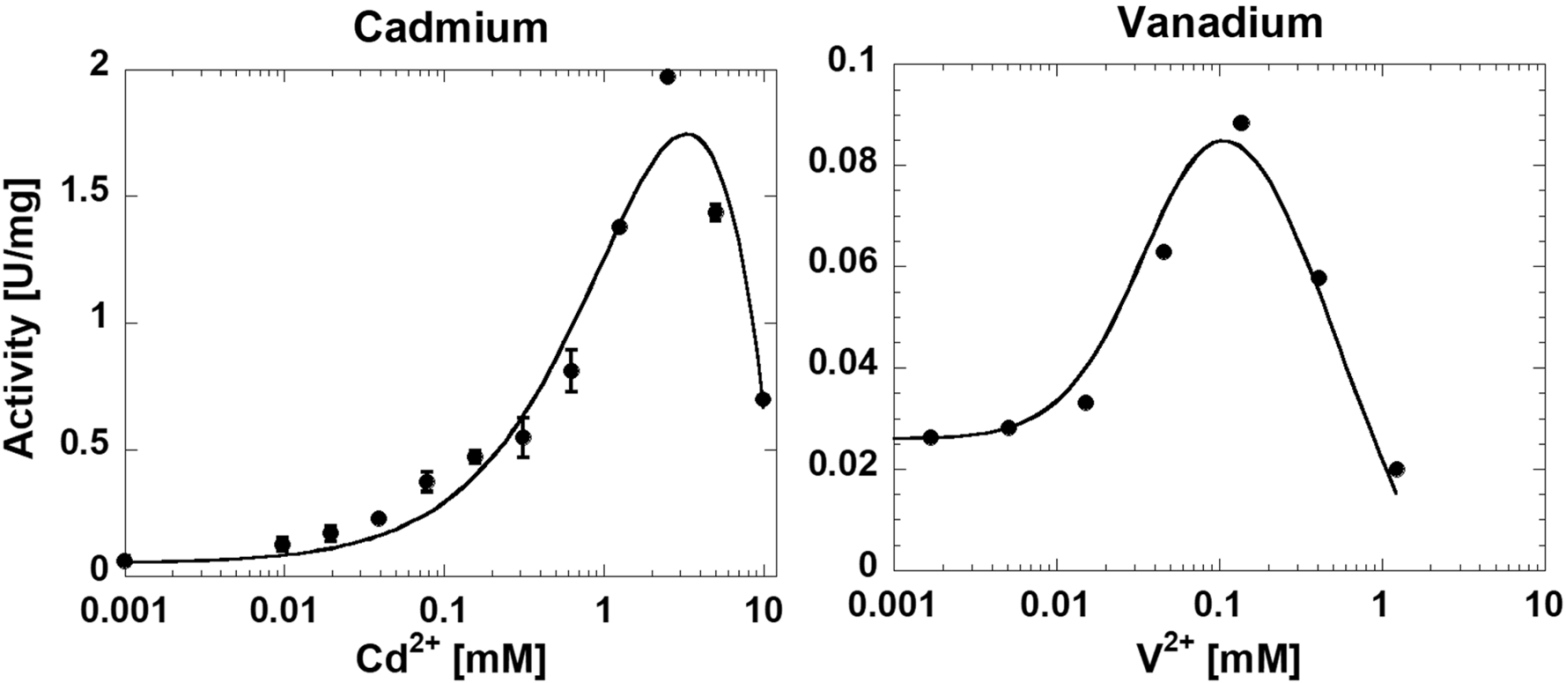
The response of hLPYK activity to changing concentrations of Cd^2+^ and V^2+^. Both Cd^2+^ and V^2+^ support low levels of hLPYK activity. For this initial screen, hLPYK purified with Mg^2+^ present was diluted into the assay cocktail, giving rise to an expected background activity due to the carryover of some small level of Mg^2+^. Error bars for Cd^2+^ data are the standard deviation for three replicate reads and when they are not visible, they are smaller than data points. The V^2+^ data was only collected once, so no error bars are included. Lines are to show data trends only. Other divalent cations that supported activity were further evaluated using a buffer containing the respective divalent cations (Mg^2+^, Mn^2+^, Co^2+^, Ni^2+^, and Zn^2+^). Cu^2+^, Pb^2+^ and Fe^2+^ supported activity but also caused a precipitate to form which precluded them from the study. Ca^2+^, Ba^2+^, Be^2+^, Cr^2+^, Hg^2+^ and Sr^2+^ did not support activity in this initial divalent cation screen.

To determine which divalent cations could support hLPYK activity, we initially conducted a screen. hLPYK was purified in and stored in a buffer that included Mg^2+^. The protein was dialyzed into the buffer without any added divalent cation. Treatment of protein samples with Chelex resin with the intent of stripping away all divalent cation caused irrecoverable loss of enzymatic activity. In this design, we recognized that Mg^2+^ binding to the protein likely resulted in Mg^2+^ carryover into the assay. Due to that Mg^2+^ carryover, we anticipated and then confirmed a low-level background activity in assays when no other divalent cation were added (*i.e*., the non-zero left y-intercept in Fig. 2). We focused on identifying when added divalent cation concentrations resulted in increased activity. Mg^2+^, Mn^2+^, Co^2+^, Ni^2+^, Zn^2+^, Cd^2+^, V^2+^, Cu^2+^, Pb^2+^ and Fe^2+^ all supported activity. Ca^2+^, Cr^2+^, Ba^2+^, Be^2+^, Hg^2+^ and Sr^2+^ did not result in any activity above the background. The responses for Cd^2+^ and V^2+^ were not further characterized (Fig. 2) because of the excessive quantity of protein that would be required due to low activity. Although Cu^2+^, Pb^2+^ and Fe^2+^ supported activity, a precipitant formed at higher PEP concentrations, precluding the generation of full response curves (data not shown). Zn^2+^ was only partially carried forward in our studies.

**Figure 3:**
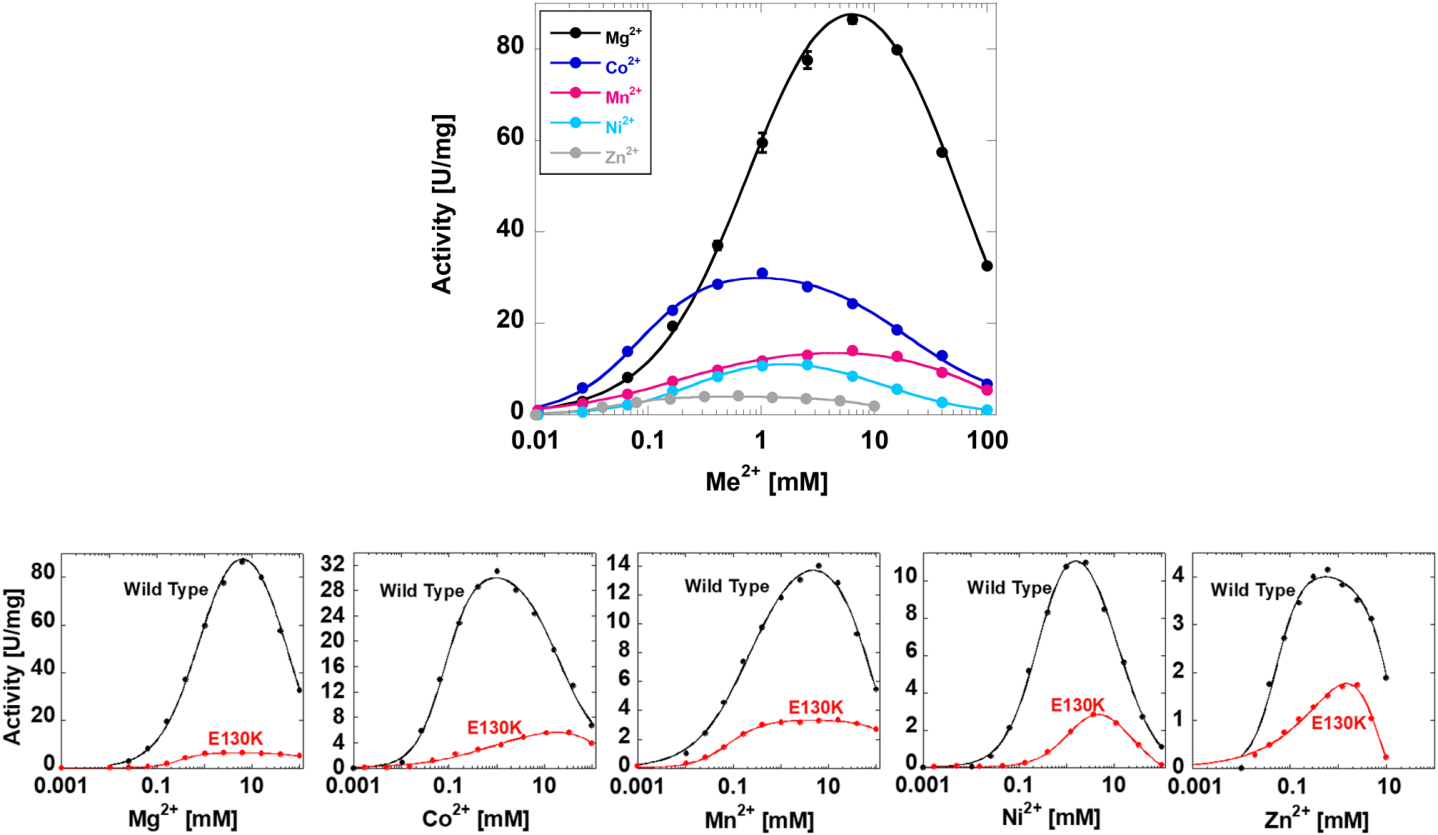
The response of hLPYK activity to varying concentrations of divalent cation. Top) Me^2+^ is used to indicate the divalent cation form of metals studied. These assays were completed in the absence of allosteric effectors. These data were collected with hLPYK purified in buffer containing the same divalent cation assayed (*i.e.,* different purifications for each divalent cation). 10 mM PEP and 2 mM ADP were used in these assays. Error bars are the standard deviation for three replicate reads and when they are not visible, they are smaller than data points. Lines are to show data trends only. Bottom) The influence of the E130K mutation on substrate inhibition to divalent cations. To test the idea that the observed substrate inhibition pattern that results from increasing divalent cation concentration is due to the divalent cation competing with K^+^ binding to the monovalent cation site, the E130K mutation was created. This mutation inserts a side-chain positive charge from lysine in the location of the monovalent cation^21^, rendering the enzyme monovalent cation independent and likely preventing the binding of any type of cation to the monovalent cation site. For all divalent cations characterized, the inhibition phase of the response is shifted to higher divalent cation concentrations for the E130K protein as compared to the wild type protein. Wild type data are the same as presented in the top panel. Error bars are the standard deviation for three replicate reads and when they are not visible, they are smaller than data points. Lines are to show data trends only.

Our next design was to repeat the response of activity to increasing divalent cation concentrations using hLPYK that was purified in the absence of added divalent cation. The same protein preparation was used to perform all subsequent assays. Consistent with the first data set, the divalent cations used in this second design supported activity. By focusing on the highest level of activity at any concentration, the order of activity (greatest to least) was Mg^2+^ > Co^2+^ > Mn^2+^ > Ni^2+^ > Zn^2+^ (Fig. 3; top panel). The concentration that resulted in the maximum activity for each divalent cations did not correlate with the order of highest activity elicited for each divalent cation.

A notable aspect of the relationship between hLPYK activity and the divalent cation types is the observed substrate inhibition pattern. This response reflects an inhibition at higher divalent cation concentrations, regardless of the divalent cation considered. A previous hypothesis for this response suggests that higher divalent cation concentrations compete with the monovalent cation resulting in inhibition^16^. To test this hypothesis, we generated the E130K mutation that substitutes the catalytically-required monovalent cation with a lysine residue^14,21^. We purified this mutant protein in the buffer without divalent cations and found that it retained activity when assayed in the presence of each of the divalent cations, albeit always less than the wild-type protein assayed under identical conditions (see Fig. 3). For each divalent cation, the substrate inhibition was at a higher divalent cation concentration in E130K compared to the wild type protein. Therefore, the data are consistent with high concentrations of divalent cations binding competitively with the monovalent cation. However, at even higher concentrations of divalent cation, an apparent substrate inhibition response persists in the E130K data that are assumed to be independent of divalent cation binding at the monovalent cation binding site.

To explain the substrate inhibition response, we first considered the possibility that one or more low-affinity divalent cation binding sites might be responsible for binding ions when present at high concentrations, thus eliciting the inhibition. These sites could function through an allosteric mechanism. However, we also recognized that other mechanisms could cause substrate inhibition response patterns. We can first acknowledge that substrate inhibition is typically attributed to the formation of dead-end complexes, some of which are specific to ordered binding mechanisms^38,39^. Because PYK is thought to bind its two substrates randomly^40,41^, this mechanism is less likely to function in the hLPYK.

**Figure 4:**
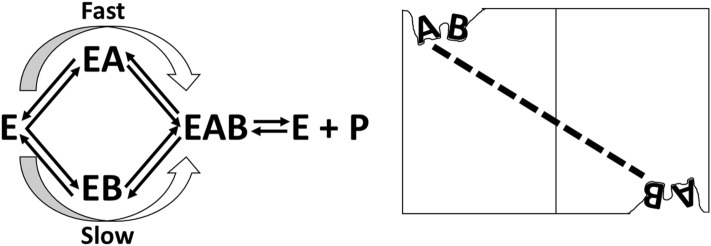
Examples of alternative mechanisms that can give rise to substrate inhibition response patterns. (Left) A kinetic mechanism in which two different orders of substrate-binding result in different rates of reaction. In this example, when A is held constant at a subsaturating concentration and B is varied, at a low concentration of B, the reaction will proceed through the fast route, but as the concentration of B increases, B is likely to bind first to result in a slower reaction^42,43^. (Right) An allosteric mechanism in which substrate A binding to one active site of a multimeric protein can act as an allosteric inhibitor of substrate B binding in a second active site. When one of the two substrates is present at subsaturating concentrations and the concentration of the second substrate is varied, a substrate inhibition response will be observed^44,45^.

Another potential kinetic mechanism to explain a substrate inhibition pattern involves two alternative ordered kinetic pathways for a two-substrate system with two different rates (Fig. 4): If substrate A binds first, the total reaction rate is fast, but if substrate B binds first the reaction is slow^42,43^. Therefore, as the concentration of B is varied when the concentration of A is held constant at a concentration much lower than the K_M_, the reaction at low B would proceed through the faster A-binding first pathway. As the concentration of the B substrate is increased, the reaction mechanism would change to use the slower B-binding first pathway, thus resulting in slower rates to result in a substrate inhibition response over the concentration range of B. If at low divalent cation concentration, the order of protein-bound divalent cation and nucleotide bound divalent cation results in different reaction rates, then this proposed mechanism could give rise to the observed substrate inhibition pattern.

A much less recognized mechanism that can generate a substrate inhibition pattern is when an active site ligand in one subunit serves as an allosteric inhibitor of a second ligand type binding in a second active site in a multimeric protein (Fig. 4)^44,45^. When varying divalent cation concentration, we used concentrations of substrates and monovalent cation (i.e. PEP, ADP, and K^+^) that were saturating, thus precluding the observation of an apparent substrate inhibition due to allosteric coupling between active site ligands. However, due to the requirement for two divalent cations in each active site, our design could not eliminate the potential that the “protein-bound” divalent cation might be allosterically coupled to the nucleotide-bound divalent cation in a second active site. Therefore, the observed substrate inhibition could result from an allosteric coupling between active site ligands binding in two different active sites of the homotetrameric hLPYK.

### Allosteric response as a function of divalent cation type

**Figure 5:**
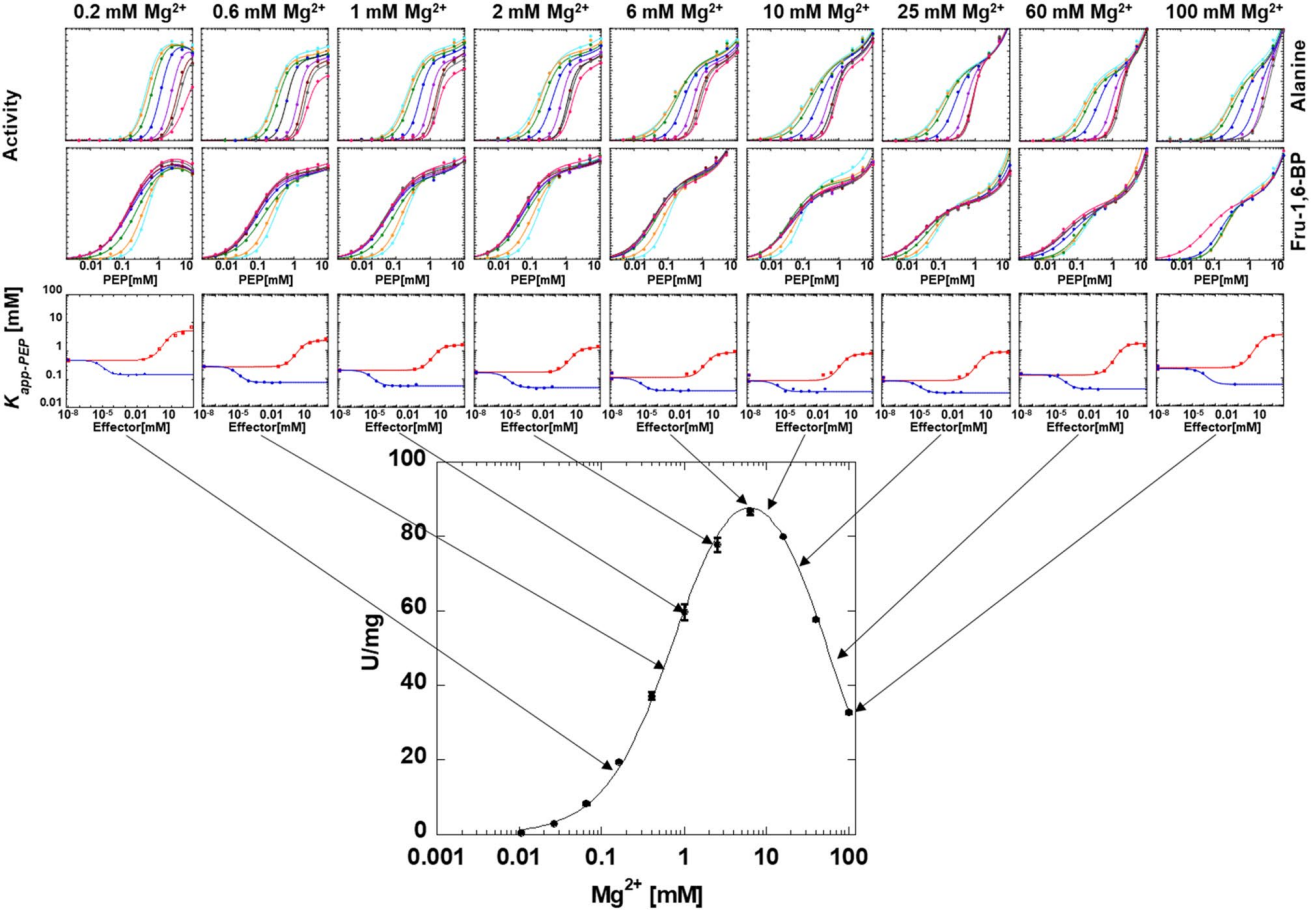
The influence of varying concentrations of Mg^2+^ on allosteric inhibition of PEP affinity by alanine and the allosteric activation of PEP affinity by Fru-1,6-BP. The response to varying concentrations of Mg^2+^ in the lower panel includes the same data from Fig. 3. Lines in the first two rows of panels represent data fits to Eq. (1). Error bars in the third row of panels represent error estimates from the parameter fits of the data in the first two rows of panels. When error bars are not visible, they are are smaller than data points. Lines in the third row of panels represent data fits to Eq. (2).

To evaluate the influence of divalent cation types and concentrations on allosteric functions, the apparent affinity of hLPYK for PEP (*K*_*app-PEP*_) was determined at a constant, saturating ADP concentration. On a graph of activity as a function of PEP concentration (top two rows of Fig. 5), the *K*_*app-PEP*_ value is determined as the concentration that results in 1/2 *V*_*max*_ activity. In addition to the expected activity response as the PEP concentration increases, a second phase in the response curve is apparent. To obtain *K*_*app-PEP*_ values, the activity dependence on PEP concentration was fit to:

1 $$v = \frac{{V_{{{\text{max}}}} \left[ {{\text{PEP}}} \right]^{{{\text{n}}_{{\text{H}}} }} }}{{\left( {K_{{\text{app - PEP}}} } \right)^{{{\text{n}}_{{\text{H}}} }} + \left[ {{\text{PEP}}} \right]^{{{\text{n}}_{{\text{H}}} }} }} + c,$$

where *V*_max_ is the maximum velocity, *K*_app-PEP_ is the concentration of substrate that yields a rate equal to one-half the *V*_max_, n_H_ is the Hill coefficient, and *c* is a slope for change at high PEP concentration^37^.

**Figure 6:**
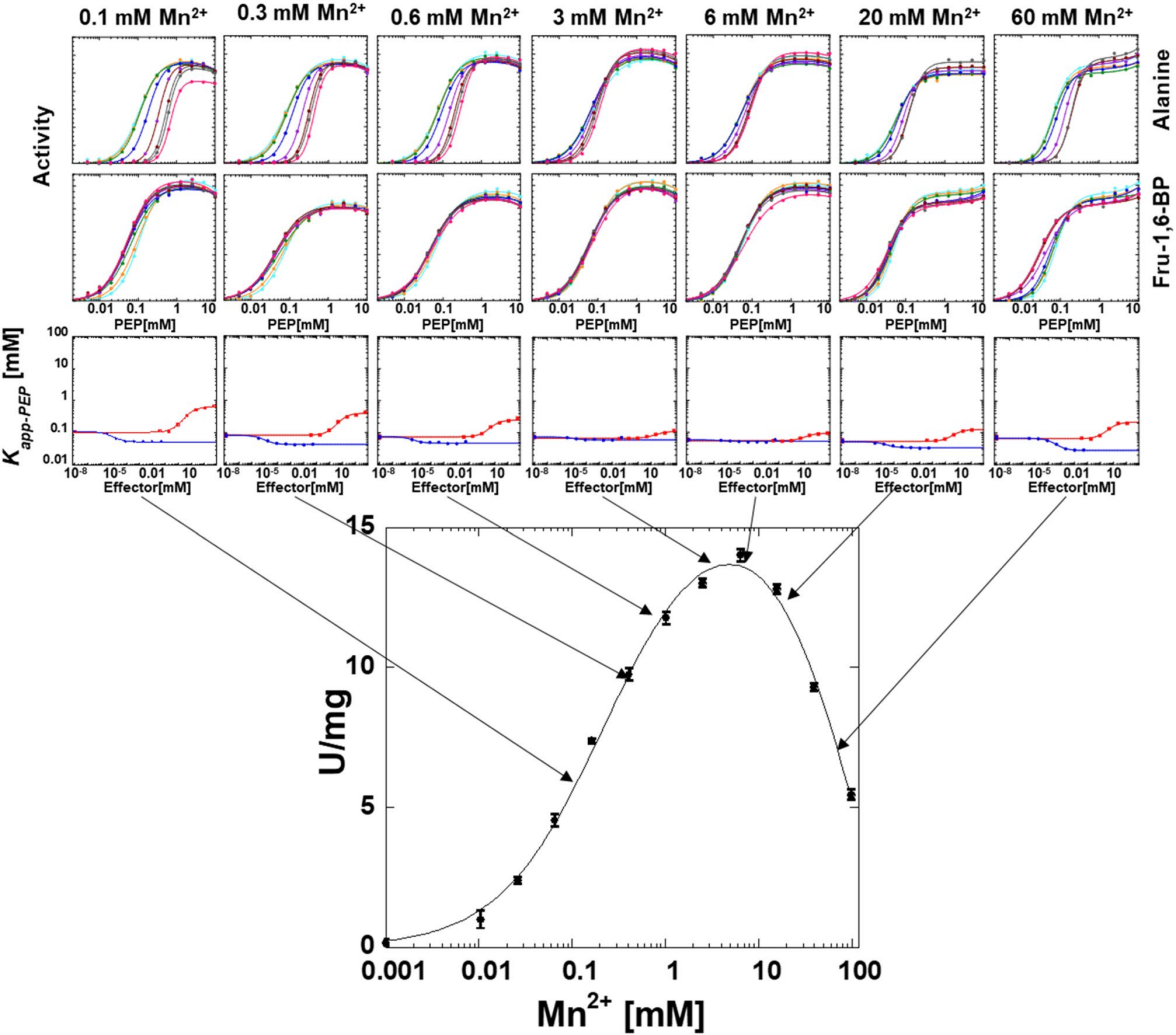
The influence of varying concentrations of Mn^2+^ on allosteric inhibition of PEP affinity by alanine and the allosteric activation of PEP affinity by Fru-1,6-BP. The response to varying concentrations of Mn^2+^ in the lower panel includes the same data from Fig. 3. Lines in the first two rows of panels represent data fits to Eq. (1). Error bars in the third row of panels represent error estimates from the parameter fits of the data in the first two rows of panels. When error bars are not visible, they are smaller than data points. Lines in the third row of panels represent data fits to Eq. (2).

**Figure 7:**
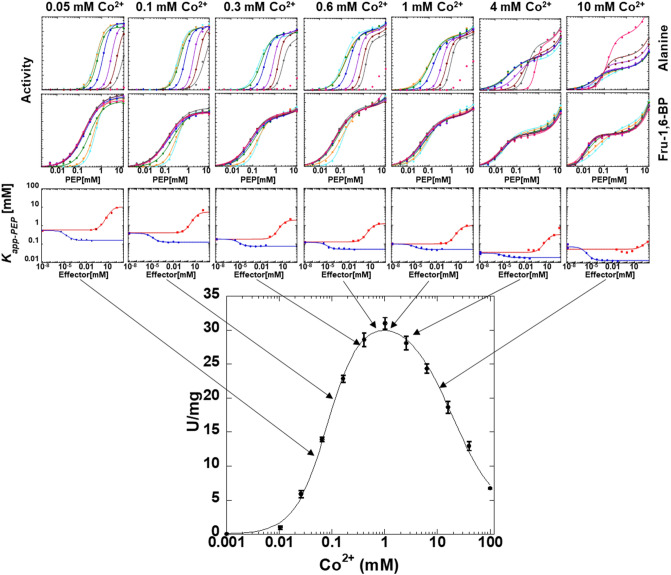
The influence of varying concentrations of Co^2+^ on allosteric inhibition of PEP affinity by alanine and the allosteric activation of PEP affinity by Fru-1,6-BP. The response to varying concentrations of Co^2+^ in the lower panel includes the same data from Fig. 3. Lines in the first two rows of panels represent data fits to Eq. (1). The activity response at the highest concentration of Ala (pink data) are not fit in all graphs (top row). Error bars in the third row of panels represent error estimates from the parameter fits of the data in the first two rows of panels. When error bars are not visible, they are smaller than data points. Lines in the third row of panels represent data fits to Eq. (2).

**Figure 8:**
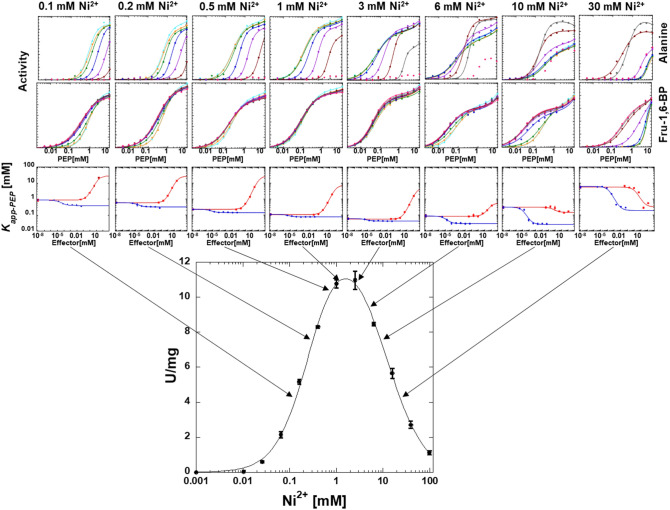
The influence of varying concentrations of Ni^2+^ on allosteric inhibition of PEP affinity by alanine and the allosteric activation of PEP affinity by Fru-1,6-BP. The response to varying concentrations of Ni^2+^ in the lower panel includes the same data from Fig. 3. Lines in the first two rows of panels represent data fits to Eq. (1). The activity response at the highest concentration of Ala (pink data) are not fit in all graphs (top row). Error bars in the third row of panels represent error estimates from the parameter fits of the data in the first two rows of panels. When error bars are not visible, they are smaller than data points. Lines in the third row of panels represent data fits to Eq. (2).

Although a second phase in the response to PEP concentration was consistent throughout our data, the nature of the second phase was a function of divalent cation concentration. At high divalent cation concentration, this second phase is an increase in activity at high PEP (Figs. 5, 6, 7 and 8). We previously provided data consistent with phosphate contamination in PEP serving as a V-type allosteric activator^37^. Only at high PEP concentrations, does that phosphate contamination become sufficient to be noted. In an alanine-scanning mutational study, there was a “patch” of positions on the surface of the protein that when replaced individually with alanine, resulted in a loss of the second phase of the response to increasing PEP concentration^46^. This led us to speculate that residues in this patch may contribute to the V-type regulation by phosphate, either as a phosphate binding site, or a central region relevant to the allosteric regulation by phosphate. At low concentrations of divalent cation (Figs. 5, 6, 7 and 8), the second phase of the PEP titration appears as substrate inhibition. This likely reflects increasing concentrations of PEP and/or contaminating phosphate binding to the constant low concentration of divalent cation to result in a reduction in free divalent cation. The result would be a lower free divalent cation concentration available to fill the protein-bound and ADP-bound requirements of the system. Although this explanation for the substrate inhibition observed in the response to varying PEP concentration at low divalent cation concentration is highly possible, other possible mechanisms for substrate inhibition (as described above for the response to varying divalent cation concentration) could also be the cause of the observed inhibition.

**Figure 9:**
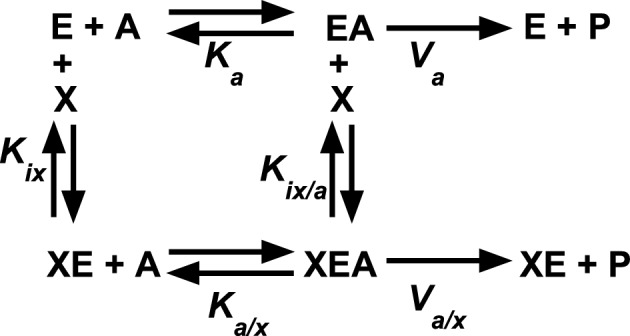
Allosteric coupling is defined by a thermodynamic energy scheme. In this reaction scheme, “E” is the enzyme that binds the substrate “A,” and allosteric effector “X.” *K*_*a*_ and *K*_*a/x*_ are the affinity values for substrate (*K*_*app*_) in the absence and presence of saturating concentrations of effector as. derived from enzymatic kinetic responses. *K*_*ix*_ and *K*_*ix/a*_ are equilibrium binding constants for the effector to the enzyme in the absence and presence of substrate, respectively.

To determine the magnitude of the observed allosteric coupling, the *K*_*app-PEP*_ was determined over a concentration range of the allosteric effector, either Fru-1,6-BP or alanine (*i.e.*, a K vs. X graph). Thus, one 96-well plate was used to evaluate one allosteric effector/PEP coupling in one K vs. X graph (12 wells to determine *K*_*app-PEP*_ at each of 8 effector concentrations). Graphs of *K*_*app-PEP*_ as a function of effector concentration were fit to:

2 $$K_{{\text{app - PEP}}} \, = \,K_{{\text{a}}} \,\left( {\frac{{K_{{{\text{ix}}}} \, + \,\left[ {{\text{Effector}}} \right]}}{{K_{{{\text{ix}}}} \, + \,Q_{{{\text{ax}}}} \,\left[ {{\text{Effector}}} \right]}}} \right),$$

where *K*_a_ = *K*_app-PEP_ when [Effector] = 0;* K*_ix_ = the dissociation constant for effector (X) binding to the protein in the absence of substrate (A); and *Q*_ax_ is the allosteric coupling constant discussed in detail elsewhere^47^. This equation is derived from a linked-binding function analysis for allosteric regulation (Fig. 9)^47,48^. These fits are used to obtain both the binding affinity of the effector (*K*_*ix-Ala*_ or *K*_*ix-FBP*_) and the pairwise coupling constant (*Q*_*ax*_) that relates the influence of the effector on the binding of the substrate to the protein^14,48^. *Q*_*ax*_ is defined (Eq. (3)) as the affinity of the enzyme for substrate in the absence *vs.* presence of the effector (*K*_*ia*_*/K*_*ia/x*_) and the affinity of the enzyme for effector in the absence *vs*. presence of substrate (*K*_*ix*_/*K*_*ix/a*_) using parameters defined in Fig. 9.

3 $${Q}_{ax}=\frac{{K}_{ia}}{{K}_{ia/x}}=\frac{{K}_{ix}}{{K}_{ix/a}}$$

Importantly, our evaluation of divalent cations in the allosteric mechanism must first divide divalent cations binding in the active site that is experiencing catalytic turnover *vs*. divalent cations binding in other active sites. Because our design monitored enzymatic activity, even at low divalent cation concentrations, the requirement for divalent cations for activity implies that any active site that supports enzymatic turnover (*i.e*., the site that contributes to determining *K*_*app-PEP*_) must have both divalent cation sites occupied. In this experimental design, the only probe for the role of the divalent cations in active sites that undergo turnover (*i.e*., that reported on PEP affinity) was different divalent cation type. Changes in divalent cation concentration were NOT a probe for the role of divalent cation within the active sites undergoing turnover. It follows that the data set generated herein does not include information for allosteric coupling in the complete absence of divalent cation, precluding any plus *vs*. minus divalent cation comparisons of allosteric coupling. As previously noted^14^, the magnitude of the allosteric responses when Mg^2+^ is replaced with Mn^2+^ is much reduced. Ni^2+^ and Co^2+^ also reduce the magnitude of the allosteric response Fru-1,6-BP. However, Ni^2+^ causes a larger allosteric response to Ala. (See additional notes on the Ni^2+^ response below).

### The responses of ligand binding affinities as a function of divalent cation concentration

**Figure 10:**
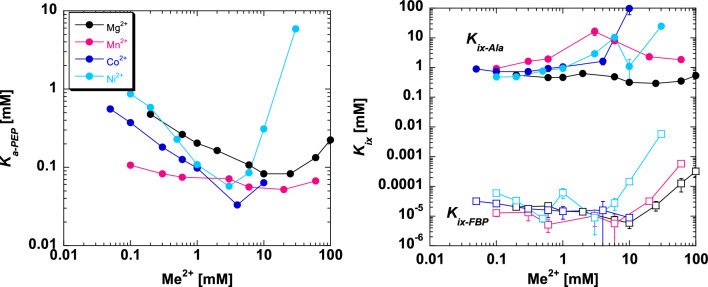
The response of three types of ligand binding to divalent cation type and concentration (Mg^2+^ black, Mn^2+^ pink; Co^2+^ royal blue; Ni^2+^ light blue). Left) *K*_*a-PEP*_ values. Right) *K*_*ix*_ values for both Ala (filled circles) and Fru-1,6-BP binding (open squares). Importantly, because data were collected by observing enzymatic response and enzymatic turnover is thought to depend on the presence of divalent cation, even at low divalent cation we anticipate that a divalent cation was present in the active site reporting function. Values were derived from Figs. 5, 6, 7 and 8 and fits to Eq. (2). Error bars represent error estimates obtained from data fits and when not visible, they are smaller than data points. Note that a reduced allosteric response makes it more challenging to evaluate *K*_*ix-Ala*_ (*i.e*., see 5 and 10 mM Ni^2+^, where the response to Ala changes from allosteric inhibitor to allosteric activator), resulting in more noise in the data trend.

The K *vs*. X data analysis was repeated across a range of divalent cation concentrations. Full data sets are provided in Figs. 5, 6, 7 and 8. Binding parameters obtained from Figs. 5, 6, 7 and 8 are plotted as a function of divalent cation concentration in Fig. 10.

For these data sets, even when PEP concentration was sufficiently low so that only one of the four active sites in the homotetramer supported activity, other active sites would have had divalent cations bound as the concentrations of divalent cation increased. Binding at the other active sites can act as modulators of activity and allosteric regulation that involves PEP in the functioning active site. Therefore, the divalent cation concentration range tested the role of divalent cations outside of the catalytic active site(s) for influence on allosteric outcomes.

In our experimental design, changes in both K^+^-PEP andK^+^-effector were matched with KCl to maintain constant K^+^. However, changes in divalent cation concentration were not matched with KCl, and that design was chosen because of the possibility that K^+^ might bind competitively in the two divalent cation binding sites. The result was that ionic strength was not constant across the divalent concentration range. *K*_*a-PEP*_ decreases (increased binding affinity) as a function of divalent cation concentrations up to ~ 5 mM. However, the reduced PEP affinity observed above 5 mM may not have been purely ionic strength given that the response was unique to each type of divalent cation, with a sharper reduction in PEP affinity as Ni^2+^ was increased above 5 mM when compared to the response to increases in Mg^2+^ and Mn^2+^. *K*_*ix-FBP*_ didn’t show much of a trend for change as divalent concentrations were increased to 5 mM, but clear reductions in affinity were observed above 5 mM, again with a sharper response for [Ni^2+^] compared to other divalent cation types.

The response of *K*_*ix-Ala*_ to increasing concentrations of divalent cation is more dependent on divalent cation type as compared to other ligand binding parameters: *K*_*ix-Ala*_ shows little response to Mg^2+^, but first increases and then decreases as a function of increasing [Mn^2+^]. The latter response is particularly intriguing. Like other biphasic responses, a new divalent cation binding site can be proposed to explain this response. However, a biphasic response can also result from a subsaturating allosteric effect.

**Figure 11:**
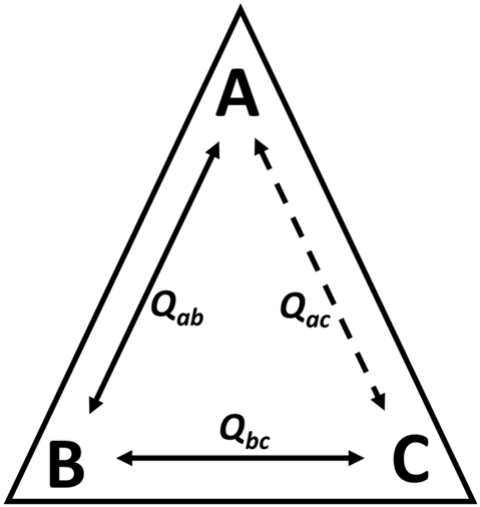
A protein (triangle) with three ligand binding sites (A, B, and C) illustrates a possible subsaturating effect. *Q*_*ac*_ is set to equal 1, so that there is no direct allosteric coupling between the binding of A and the binding of C. If *Q*_*ab*_ and *Q*_*bc*_ are both activating, then at subsaturating concentrations of B (*i.e*., partial occupancy of the B site), the binding of C will increase the occupancy of B, which in turn will result in allosteric activation of A binding. If *Q*_*ab*_ and *Q*_*bc*_ are both inhibiting, then at subsaturating concentrations of B (*i.e*., partial occupancy of the B site), the binding of C will remove bound B, which in turn, removes allosteric inhibition of A binding. Thus, in both scenarios, at subsaturating concentrations of B, increases in the concentration of C appear to allosterically activate the binding of A, despite that *Q*_*ac*_ is equal to 1. No allosteric responses of A binding to the presence of C are expected when B is absent or at a concentration sufficiently high to saturate the B binding site. Therefore, across a concentration range of B, the influence of C on A binding would go from no influence at low [B], to activation at intermediate [B], to no influence at high [B]^49–51^.

The thoughts behind a subsaturating effect were initiated by Weber^49,50^ and further detailed by Reinhart^51^. To understand the subsaturating effect, consider a protein that has three binding sites that each bind chemically unique ligands A, B, and C (Fig. 11). Three pairs of allosteric coupling events include coupling between A and B, between B and C, and between A and C. We will assume that there is no allosteric coupling between A and C, such that *Q*_*ax*_ = 1. When B is absent, the addition of C will not influence the binding of A. When B is at a sufficiently high concentration to saturate the B binding site, the addition of C will not influence the binding of A. That leaves us to consider the case when site B is only partially occupied (a subsaturating concentration of B). In the scenario when *Q*_*ab*_ and *Q*_*bc*_ are both greater than 1 (i.e. activating), then when C is added, the binding affinity for B will be increased and result in higher occupancy of the B binding site. Increased B occupancy will, in turn, result in allosteric activation of A binding. Therefore, the apparent response at this subsaturating concentration of B will be that the addition of C results in increased binding affinity for A. In the case where *Q*_*ab*_ and *Q*_*bc*_ are both inhibiting (i.e. less than 1) and then when C is added, the binding affinity for B will be reduced and lower occupancy of the B binding site. However, this equates to a removal of an allosteric inhibitor, such that A binding can bind with higher affinity. Therefore, whether *Q*_*ab*_ and *Q*_*bc*_ are both activating or both inhibiting, the addition of C when B is subsaturating will result in increased A binding. It follows that the expected response of additions of C to alter A binding across a concentration range of B is; (1) no influence of C on A binding in the absence of B; (2) activation of A binding by the addition of C at an intermediate concentration of B; and (3) no influence of C on A binding when the concentration of B is high. Although assuming *Q*_*ac*_ = 1 helps introduce the topic, in fact, the same subsaturating effect can be present when there is allosteric coupling between A and C, with the anticipated outcome that *Q*_*ac*_ starts at some value, increases at intermediate concentrations of B, and then returns to the initial value at high concentrations of B. That scenario is observed in Fig. 10 for the *K*_*ix-Ala*_ response to increasing [Mn^2+^]. Therefore, a subsaturating effect must also be considered along-side the possibility that at higher concentrations of divalent cation, distinct lower-affinity divalent cation binding sites are being filled and eliciting a response.

### The responses of allosteric coupling as a function of divalent cation type

**Figure 12:**
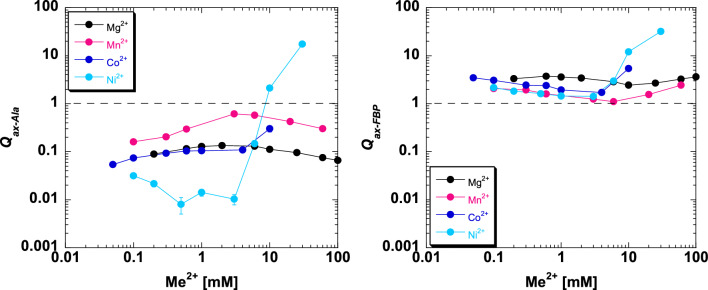
The response of *Q*_*ax-FBP*_ and *Q*_*ax-Ala*_ to divalent cation type and concentration. Left) *Q*_*ax-Ala*_ responses. Right) *Q*_*ax-FBP*_ responses. Importantly, because data were collected by observing enzymatic response and enzymatic turnover is thought to depend on the presence of divalent cation, even at low divalent cation we anticipate that a divalent cation was present in the active site reporting function. Values were derived from Figs. 5, 6, 7 and 8 and fits to Eq. (2). Error bars represent error estimates obtained from data fits and when not visible, they are smaller than data points. Due to Eq. (3), the lack of an allosteric response is *Q*_*ax*_ = 1, indicated by a horizontal dashed line. *Q*_*ax*_ values above 1 indicate allosteric activation and values below 1 indicate allosteric inhibition.

Allosteric coupling constants obtained from Figs. 5, 6, 7 and 8 are plotted as a function of divalent cation concentration in Fig. 12. The allosteric coupling constants also show data trends to indicate that the response is not due purely to increasing ionic strength. The ~ 5 mM concentration is again a divider for two data trends for *Q*_*ax-Ala*_ and *Q*_*ax-FBP*_. *Q*_*ax-Ala*_ increases in response to [Mg^2+^], [Mn^2+^], and [Co^2+^] up to ~ 5 mM. Above 5 mM, increasing [Mg^2+^] and [Mn^2+^] caused a reduction in *Q*_*ax-Ala*_. *Q*_*ax-FBP*_ decreased as concentrations of divalent cations is increased, up to the ~ 5 mM concentration, but increased at higher concentrations of divalent cations. Again, the potential of additional divalent cation sites and/or the subsaturating allosteric effect could contribute to these observed data trends.

### The response to Ni^2+^ may imply a unique mechanism

**Figure 13:**
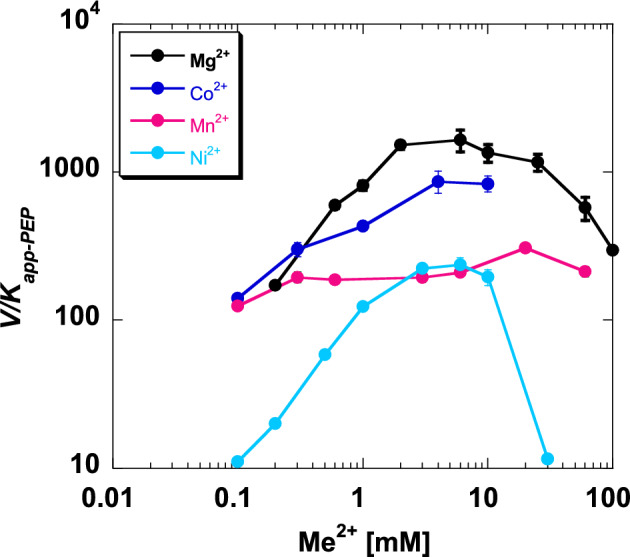
*V/K* data, where K indicates *K*_*app-PEP*_. Values were taken from data collection in Figs. 5, 6, 7 and 8 at zero effector. Lines connecting dots are used to indicate data trends. Error bars represent errors taken from fit estimates.

At the onset of this study, we had hoped to identify data trends that were dependent on some properties of ions used (e.g. ionic radius, charge density). We have not identified that type of correlation. In fact, we retrospectively also considered the V/K as a function of divalent cation concentration (Fig. 13). These responses for each ion type may be sufficiently different to support the suggestion that a different microscopic reaction step is rate-limiting for each different divalent cation type (i.e. different kinetic mechanisms). A role for changes in individual microscopic rate constants as an allosteric mechanism has been evaluated both in the context of whether on-rates *vs.* off-rates were modified to alter substrate binding, and in the context of altered product-release to give rise to a V-type allosteric response^52–54^. Therefore, it stands to reason that if different divalent cation types do indeed cause different microscopic reaction steps in the catalytic mechanism to be rate limiting, then the allosteric mechanisms would be unique to the divalent cation type.

When compared to other divalent cation types, the response to increasing Ni^2+^ shows several unique properties. In Fig. 8, at the highest concentrations of Ni^2+^, a substrate inhibition response is apparent in the response to PEP titrations. That type of substrate inhibition is not observed in the presence of any other of the divalent cations studied. At the highest concentrations of Ni^2+^ used in our study, alanine transitions from being an inhibitor of PEP affinity to being an allosteric activator that promotes higher PEP affinity. (Data trends with Co^2+^ could be consistent with Ala being an activator if even higher concentrations of Co^2+^ were to be included.) The responses of ligand binding (Fig. 10) and allosteric coupling constants (Fig. 12) to increases in [Ni^2+^] above the ~ 5 mM division result in a sharper transition than for other divalent cations. Taken together with the knowledge that Ni^2+^ can form complexes with several small molecules, these data trends may indicate that a Ni-complex interacts and uniquely influences the function of hLPYK, compared to other divalent cations.

## Conclusions

As the field becomes more interested in evaluating how additional ligands alter allosteric responses between a substrate and an allosteric effector, graphing *Q*_*ax*_ as a function of a third ligand concentration offers a method to visualize that effect. In contrast, three-dimensional graphs like those used in the evaluation of divalent cation influence on yeast PYK allosteric regulation^24–27^ are challenging to visualize when substrate inhibition responses are intrinsic to the system.

Although the complex equilibrium highlighted in this study may be represented in allosteric regulation of many other kinases that use divalent cations in the active site mechanisms, there are also likely systems that bind more than two ligands and lack the complex equilibrium component of the hLPYK system. Simpler mechanisms with reduced complex equilibrium challenges may be more amenable for obtaining quantitative data fits. However, by starting with the consideration of many potential mechanisms that can give rise to each type of observation, as listed in this work, new experimental designs can be considered to eliminate potential mechanisms (*e.g*. hybrid tetramers to distinguish between the kinetic and allosteric origins of substrate inhibition^44^) on the pathway to elucidating the true mechanism associated with various responses. In particular, correlations of functional response curves with protein biophysical signals (e.g. NMR or Förster Resonance Energy Transfer) are likely to be useful in distinguishing when a given response is derived from changes in the protein itself.

## Materials and methods

### Cell growth and protein expression

The protein was expressed as 6His-MBP-SUMO-hLPYK (MBP stands for maltose binding protein) in the pCDFDuet-1 (Novagene) in the QTF60 strain of *E. coli*^55^. The E130K mutation was added via QuikChange mutagenesis (Agilent). For both wild type and the E130K mutant, overnight LB cultures (including 100 µg/mL Spectinomycin and 5 mM lactose to induce protein expression) were grown at 37 °C and with shaking at 225 rpm for 20 h. Cells were harvested via centrifugation at 8000 rpm in an SLA-3000 rotor for 20 min.

### Protein purification

The cell pellet was resuspended in a minimal volume of the spent media supernatant, transferred to a 50 mL conical centrifuge tube, and pelleted at 4200 rpm for 20 min using a Sorvall Legend XTR benchtop centrifuge with a TX-1000 rotor. The supernatant was poured off and the cell pellet was stored at -20 °C until used. The purification buffer used throughout was 10 mM MES (pH 6.8), 10 mM KCl, and 10 mM TCEP; TCEP was always added fresh immediately before use, followed by an adjustment of the pH. The cell pellet was minimally thawed on ice (only until the frozen pellet would break away from the tube; further delay reduces yield) and then suspended in purification buffer with 25% (v/v) glycerol and brought to a total volume of 32.5 mL. The suspension was sonicated in ice water for 4 min at 100% amplitude (5 s sonication on, 45 s off). Cell debris was removed via centrifugation at 14000 *rpm* in an SS-34 rotor for 90 min. 10 mL of amylose resin (New England Biolabs) was prepared and equilibrated with 20 column volumes (CV) using de-gassed purification buffer. The clarified protein sample was loaded onto the amylose column and washed with 30 CVs of purification buffer. Subsequently, the MBP tagged protein was eluted with purification buffer + 10 mM Maltose. Fractions with PYK activity were pooled and an in-house-purified SUMO-protease^56,57^ was added to cleave the 6His-MBP tag. The solution was left overnight at 4 °C for optimal cleavage. After overnight cleavage, the sample was dialyzed for 2 h with 3 buffer exchanges in purification buffer using 12–14 kDa dialysis tubing that was previously boiled in ethanol and EDTA and thoroughly rinsed with 18-Ω water. Ammonium sulfate precipitation (0.267 g/mL (NH_4_)_2_SO_4_) was used to remove the cleaved hLPYK protein from uncleaved tagged hLPYK, the cleaved 6His-MBP-SUMO tag, and the SUMO protease, while simultaneously concentrating the cleaved hLPYK protein. Precipitated, purified, tag-cleaved hLPYK was recovered by centrifugation at 14000 *rpm* for 1 h using an SS-34 rotor. The pellet was resuspended in ~ 400 μL of purification buffer. The protein was dialyzed for an additional 2 h (3 exchanges) using purification buffer without divalent cation. Protein concentration in mg/ml was determined by measuring A_280_ and dividing by 0.5 mL mg^−1^ cm^−1^^12^.

### Protein freezing, storing, and thawing

Recovery of pre-frozen wild type protein properties (mainly preventing a freeze/thaw dependent drift in *K*_*a-PEP*_) was equally sensitive to the freezing protocol as to the thawing protocol. For freezing, purified protein in purification buffer was divided into 50µL aliquots in thin-walled PCR-tubes and were flash-frozen by plunging the tube into liquid nitrogen. The frozen protein was stored at − 80 °C. When thawed, the aliquot was immersed in a 42 °C water bath until the last visible signs of ice disappears then the sample tube was immediately put on ice.

### Enzyme assays and data fitting

PEP binding affinity and the allosteric response were measured for the various divalent cations that supported activity, and for each divalent metal, a range of concentrations was assessed. A coupled enzyme assay with LDH (in excess) was used. The assay detects the consumption of NADH, resulting in a decrease in absorbance at 340 nm. The assays were carried out as previously described^37^, in 50 mM HEPES (pH 7) at 30 °C, 2 mM ADP, and varied divalent cation types over a range of concentrations. The pH of the assay buffer was adjusted with KOH and HCl. Total K^+^ derived from the adjustment of pH and as counterions from any buffer/assay components were summed and supplemented by the addition of KCl to result in a final K^+^ of 350 mM in the final assay. The assay cocktail and effectors were thoroughly mixed and incubated at 30 °C before initiating the assay with PEP. Assays were performed using a 96-well plate. A SpectraMax M5 spectrophotometer (Molecular Devices) was used to read absorbance at 340 nm over time.

## Supplementary Information

Supplementary Table 1.

Supplementary Information.

## Data Availability

All data generated in this study are included in the supplemental information. Supplemental Information includes: **(**1) A table of many of the possible binding interactions; (2) Both raw data and data fits generated in this study.
