# Supplementary material for: Divalent cations in human liver pyruvate kinase exemplify the combined effects of complex-equilibrium and allosteric regulation
Source: Sci Rep. 2023 Jun 29;13:10557. doi: 10.1038/s41598-023-36943-2 (PMC10310847; doi:10.1038/s41598-023-36943-2)
Supplement: Supplementary file 1 — Supplementary Table 1. [file 41598_2023_36943_MOESM1_ESM.pdf]

Supplemental Information

Divalent cations in human liver pyruvate kinase exemplify the combined effects of complex-equilibrium and allosteric regulation.

Tyler A. Martin[1] and Aron W. Fenton[1,*]

**Table 1**: A partial list of binding reactions that are relevant to the study of each divalent cation type	
Reaction #: description	Reaction Scheme
#1: Enzyme (E) to divalent cation (The "protein bound cation site" as defined in **Fig 1**.)	$E + Me^{2+} \rightleftharpoons E\text{-}Me_1$
#2: Enzyme (E) to divalent cation in nucleotide site	$E + Me^{2+} \rightleftharpoons E\text{-}Me_2$
#3: $E\text{-}Me_1$ to divalent cation in nucleotide site	$E\text{-}Me_1 + Me^{2+} \rightleftharpoons E\text{-}Me_1/Me_2$
#4: Enzyme (E) to monovalent cation	$E + K^+ \rightleftharpoons E\text{-}K$
#5: $E\text{-}Me_1$ to monovalent cation	$E\text{-}Me_1 + K^+ \rightleftharpoons E\text{-}Me_1/K$
#6: $E\text{-}Me_2$ to monovalent cation	$E\text{-}Me_2 + K^+ \rightleftharpoons E\text{-}Me_2/K$
#7: $E\text{-}Me_1/Me_2$ to monovalent cation	$E\text{-}Me_1/Me_2 + K^+ \rightleftharpoons E\text{-}Me_1/Me_2/K$
#8: E to divalent cation binding in the monovalent cation site	$E + Me^{2+} \rightleftharpoons E\text{-}Me_3$
#9: $E\text{-}Me_1$ to divalent cation binding in the monovalent cation site	$E\text{-}Me_1 + Me^{2+} \rightleftharpoons E\text{-}Me_1/Me_3$
#10: $E\text{-}Me_2$ to divalent cation binding in the monovalent cation site	$E\text{-}Me_2 + Me^{2+} \rightleftharpoons E\text{-}Me_2/Me_3$
#11: $E\text{-}Me_1/Me_2$ to divalent cation binding in the monovalent cation site	$E\text{-}Me_1/Me_2 + Me^{2+} \rightleftharpoons E\text{-}Me_1/Me_2/Me_3$
#12: ADP to divalent cation	$ADP + Me^{2+} \rightleftharpoons MeADP$
#13: ADP to monovalent cation	$ADP + K^+ \rightleftharpoons K\text{-}ADP$
#14: PEP to divalent cation	$PEP + Me^{2+} \rightleftharpoons MePEP$
#15: PEP to monovalent cation	$PEP + K^+ \rightleftharpoons K\text{-}PEP$
#16: Fru-1,6-BP to divalent cation	$FBP + Me^{2+} \rightleftharpoons MeFBP$
#17: Fru-1,6-BP to monovalent cation	$FBP + K^+ \rightleftharpoons K\text{-}FBP$
#18: Ala to divalent cation	$Ala + Me^{2+} \rightleftharpoons MeAla$
#19: Ala to monovalent cation	$Ala + K^+ \rightleftharpoons K\text{-}Ala$
#20: contaminating phosphate to divalent cation	$PO_4^{2-} + Me^{2+} \rightleftharpoons MePO_4$
#21: contaminating phosphate to monovalent cation	$PO_4^{2-} + K^+ \rightleftharpoons K_2PO_4$
#22: HEPES to divalent cation	$HEPES + Me^{2+} \rightleftharpoons MeHEPES$
#23: HEPES to divalent cation	$HEPES + K^+ \rightleftharpoons K\text{-}HEPES$
#24: $E\text{-}Me_1/K$ to PEP	$E\text{-}Me_1/K + PEP \rightleftharpoons E\text{-}Me_1/K/PEP$
#25: $E\text{-}Me_1/K$ to ADP	$E\text{-}Me_1/K + ADP \rightleftharpoons E\text{-}Me_1/K/ADP$
#26: $E\text{-}Me_1/K$ to MeADP	$E\text{-}Me_1/K + MeADP \rightleftharpoons E\text{-}Me_1/Me_2/K/ADP$
#27: $E\text{-}Me_1/K/MeADP$ to PEP	$E\text{-}Me_1/Me_2/K/ADP + PEP \rightleftharpoons E\text{-}Me_1/K/Me_2/ADP/PEP$
#28: $E\text{-}Me_1/K/PEP$ to MeADP	$E\text{-}Me_1/K/PEP + MeADP \rightleftharpoons E\text{-}Me_1/Me_2/K/ADP/PEP$
#29: $E\text{-}Me_1/K$ to Ala	$E\text{-}Me_1/K + Ala \rightleftharpoons E\text{-}Me_1/K/Ala$
#30: $E\text{-}Me_1/K/Ala$ to PEP	$E\text{-}Me_1/K/Ala + PEP \rightleftharpoons E\text{-}Me_1/K/Ala/PEP$
#31: $E\text{-}Me_1/K/Ala$ to ADP	$E\text{-}Me_1/K/Ala + ADP \rightleftharpoons E\text{-}Me_1/K/Ala/ADP$
#32: $E\text{-}Me_1/K/Ala$ to MeADP	$E\text{-}Me_1/K/Ala + MeADP \rightleftharpoons E\text{-}Me_1/Me_2/K/Ala/ADP$
#33: $E\text{-}Me_1/Me_2/K/Ala/ADP$ to PEP	$E\text{-}Me_1/Me_2/K/Ala/ADP + PEP \rightleftharpoons E\text{-}Me_1/Me_2/K/Ala/ADP/PEP$

#34: E-Me$_1$/K/Ala/PEP to MeADP	E-Me$_1$/Me$_2$/K/Ala/PEP + MeADP $\rightleftharpoons$ E-Me$_1$/Me$_2$/K/Ala/ADP/PEP
#35: E-Me$_1$/K to Fru-1,6-BP	E-Me$_1$/K + FBP $\rightleftharpoons$ E-Me$_1$/K/FBP
#36: E-Me$_1$/K/FBP to PEP	E-Me$_1$/K/FBP + PEP $\rightleftharpoons$ E-Me$_1$/K/FBP/PEP
#37: E-Me$_1$/K/FBP to ADP	E-Me$_1$/K/FBP + ADP $\rightleftharpoons$ E-Me$_1$/K/FBP/ADP
#38: E-Me$_1$/K/FBP to MeADP	E-Me$_1$/K/FBP + MeADP $\rightleftharpoons$ E-Me$_1$/Me$_2$/K/FBP/ADP
#39: E-Me$_1$/Me$_2$/K/FBP/ADP to PEP	E-Me$_1$/Me$_2$/K/FBP/ADP + PEP $\rightleftharpoons$ E-Me$_1$/Me$_2$/K/FBP/ADP/PEP
#40: E-Me$_1$/K/FBP/PEP to MeADP	E-Me$_1$/Me$_2$/K/FBP/PEP + MeADP $\rightleftharpoons$ E-Me$_1$/Me$_2$/K/FBP/ADP/PEP
-Reactions not explicitly listed: monovalent cation binding in defined divalent cation sites; nonspecific binding of any ligand to the protein; interactions between one type of small molecule complex and a second type of small molecule complex.<br>-FBP used in place of Fru-1,6-BP in reactions.<br>-In many entries, no effort was made to write chemically balanced reactions.	
